# Among New Books and Publications

**Published:** 1900-01

**Authors:** 


### AMONG NEW BOOKS AND PUBLICATIONS.

PERHAPS of all veterinary literature provided for the practitioner or student there is no field so poorly supplied as in the pathology of sheep. So little is taught of them in our veterinary colleges that many graduate without any knowledge beyond that of knowing that the ovine family is within his domain and that scabies and foot-and-mouth disease are scourges of this industry and field of investment. More than this, the average student has little or no knowledge of the varieties of sheep, their habits, or diseases; the vast moneyed interests wrapped up and the large number of people interested in their development and improvement. These conditions have made opportune the publication of such a work as has just been sent forth from the pen of Veterinarian William A. Rushworth, Inspector of the Bureau of Animal Industry. It will prove a work of inestimable value to the veterinary profession, not that it is destined to shed any special light on the pathology of many of the more serious scourges of sheep-breeding and raising, but that it has in a very complete manner covered the whole range of the sheep field—first, in the complete description of all the varieties of sheep, their points of merit and value, their peculiarities; second, the whole range of their many diseases, with plain statements as to treatment; third, in the compilation of all the laws relative to Federal and State inspection, importation, and exportation. The thorough manner in which this industry as a whole has been treated is bound to awaken a deeper interest in its importance as a much neglected branch of the field of veterinary medicine, and it is to be hoped that the book will meet such a reception at the hands of the profession as to insure the continued interest of the author and compiler, that its value in the field of pathology may be added to from time to time.

ANTISEPTIC TREATMENT OF WOUNDS. By DR. H. FRICK, County Veterinarian of Hettstedt, Germany. Translated and published by ALEX. EGER, Chicago, Ill. With annotations by Professors A. H. BAKER and L. A. MERILLAT.

THIS subject, which has undergone so radical a change during the past five years and made possible surgical interference along lines heretofore considered too dangerous, has awakened a wide interest among veterinarians, and, like their brother surgeons of the human family, are no longer free from the responsibility of giving that detailed attention to wounds and broken surfaces which are adopted in human surgery.

The book is divided into nine chapters, covering the history of wound-care, infection of wounds, asepsis, antisepsis, and disinfection; disinfection of infected carriers and the wound; suturing and ligation material, drainage, bandages; antiseptic mode of treating wounds; antiseptic outfit for practice; examination of wounds and their preparation for operation; operations with antiseptic precautions. In this manner it well covers the whole field of wound-care and giving the best methods, latest treatment of the subject, and relative value of the various remedies used in the treatment of wounds. It will prove an addition to veterinary literature that should be in the hands of a large number of practitioners, whose carelessness and neglect of every precautionary measure lead to much loss of time, money, and service in tedious wound-healing that have made unpopular many lines of surgical interference that should be more generally adopted. This book will be a revelation to many of such members of the profession.

MANUAL OF PRESCRIPTION WRITING AND DOSOLOGY. Prepared for Students and Practitioners of Veterinary Medicine. By H. D. HANSON, D.V.S., of New York City.

THIS, the second work of Dr. Hanson, and which has been in contemplation and preparation for some time, has just reached us. It has been dedicated by the author to Professor M. H. McKillip, founder of the McKillip Veterinary College of Chicago, as a fitting testimonial to his modest support and deep interest in the welfare of the veterinary profession.

Divided into ten chapters, covering fully prescription writing, grammatical prescription construction, Latin terms and abbreviations; combinations of drugs, and incompatabilities, solubility of drugs; weights and measures, officinal preparations, tables of doses, to which are added a large number of favorite prescriptions of prominent veterinarians. From the purely veterinary stand-point this subject has never been so completely treated of, and it will prove a helpful assistant to the student, while the older practitioner, grown careless and indifferent as to his prescription writing, will do well to spend an hour or two each week delving into its valuable contents. There will be a wide sale of this work because of its containing many favorite prescriptions of successful practitioners, and we trust it will well repay the author, who has spent so much time in the preparation of his contributions to veternary literature.

GUIDE TO PRACTICAL MEAT-INSPECTION. By DR. F. FISCHOEDER, late Director of the City Abattoirs and Stock-yards, Bromberg, Germany. Translated by A. T. PETERS, D.V.M., Investigator of Animal Diseases, Nebraska Experiment Station. Published by Alex. Eger, Chicago, Ill.

THE translator of this work, well known to the members of the American Veterinary Medical Association, has endeavored in the production of this work to translate only those parts of the author that cover in general the subject of meat-inspection, and special laws and ordinances of Germany not applicable to the United States have been supplanted by the laws and ordinances under which this work is carried on in our country.

The subject has become one of such great importance in our land that everything written or printed in the English language has been eagerly sought and studied. Our national meat-inspection service has grown more rapidly than our men have been properly equipped for the work to do.

After treating in a general way of the construction and functions

ARMY MEDICAL LIBRARY
WASHINGTON, D. C. U. S. A.

of the various parts of the animal body, the normal characters of the organs are explained at considerable length and made clearer by numerous illustrations. The whole field of instructions to our United States inspectors is added, as well as many valuable points from those long experienced in our meat-inspection service. Contagious and parasitic diseases are fully covered, as well as special chapters relating to the detection of trichinosis. The work will be generously received by the veterinary profession at this time and its value duly appreciated. The work deserved more careful preparation at the hands of the publisher and more efficient proofreading before sending it forth from the press.

